# Patient Awareness, Prevalence, and Risk Factors of Chronic Kidney Disease among Diabetes Mellitus and Hypertensive Patients at Jimma University Medical Center, Ethiopia

**DOI:** 10.1155/2019/2383508

**Published:** 2019-05-12

**Authors:** Kabaye Kumela Goro, Amare Desalegn Wolide, Fantu Kerga Dibaba, Fanta Gashe Fufa, Aster Wakjira Garedow, Birtukan Edilu Tufa, Eshetu Mulisa Bobasa

**Affiliations:** ^1^School of Pharmacy, Health Science Institute, Jimma University, Jimma, Ethiopia; ^2^School of Biomedical Science, Health Science Institute, Jimma University, Jimma, Ethiopia; ^3^School of Midwifery and Nursing, Health Science Institute, Jimma University, Jimma, Ethiopia

## Abstract

**Background:**

There is an alarming rise of chronic kidney disease (CKD) prevalence globally, which is associated with significant morbidity and mortality necessitating special attention as one of the major public health problems. The burden of CKD disproportionately impacts low-income countries like Ethiopia where hypertension and diabetes mellitus, the two most important risk factors for CKD growth rate, are greatest.

**Objective:**

The aim of this study is to assess patient awareness, prevalence, and risk factors of chronic kidney disease among hypertensive and diabetes mellitus patients.

**Methods:**

Hospital based cross-sectional study design was conducted at Jimma University Medical Center among adult (≥18 years) hypertensive and diabetes mellitus patients. Informed written consent was obtained from each participant and data was collected by interview and chart review; blood and urine samples were collected for CKD screening. Glomerular filtration rate (GFR) was estimated from serum creatinine using CKD epidemiology collaboration (CKD-EPI) equation, and CKD was defined using estimated GFR (e-GFR) and albuminuria. Multivariate logistic regression was used to identify independent predictors of CKD and* p*-value <0.05 considered statistically significant.

**Result:**

Mean (±SD=standard deviation) age of participants was 54.81 ± 12.45 years and 110 (52.9%) of them were male. Only 59 (28.4%) of the participants had awareness about CKD and its risk factors. The prevalence of CKD was 26% (95% CI; 20.3%-31.8%). Factors associated with chronic kidney disease were uncontrolled blood pressure (adjusted odds ratio (AOR)=2.22,95% CI=1.01-4.76), fasting blood sugar ≥ 150 mg/dl, (AOR=3.70,95% CI=1.75-7.69), angiotensin converting enzyme inhibitors (ACEIs) nonusers, (AOR=4.35 ,95% CI=1.96-10.0), poor knowledge of CKD (AOR=3.69, 95% CI=1.48-9.20), and long duration of hypertension (AOR=4.55, 95%CI=1.72-11.11).

**Conclusion:**

Our study found out low level of patient awareness and high prevalence of CKD. The predictors of CKD were uncontrolled blood pressure, fasting blood sugar> 150 mg/dl, long duration of hypertension, ACEIs nonusers, and poor knowledge about CKD.

## 1. Introduction

Chronic kidney disease (CKD), is defined as a progressive loss of kidney function occurring over several months to years; it is characterized by the gradual replacement of normal kidney structure with fibrotic tissues [1]. When these structural changes become conspicuous, it results in decreased kidneys' ability to process waste in the blood and perform other functions. During early stages patients may present with normal or slight decrease in Glomerular filtration rate (GFR) and Albuminuria; later it progresses, leading to end-stage renal disease (ESRD) or kidney failure [1–4]. ESRD is irreversible and fatal, unless treated by dialysis or kidney transplant [5].

There is an alarming rise of CKD prevalence globally associated with significant morbidity and mortality, necessitating special attention as one of the growing public health problems. The prevalence of CKD in the general population is 13.4% globally [6] while pooled prevalence of CKD is 10.1% in the general population, 24.7% in hypertensive, and 16.6% among diabetes mellitus patients in Africa. [7]. According to the global burden of disease 2015 study, CKD was the 17^th^ leading cause of global years loss of life and one of the fastest rising major causes of death because overall mortality due to CKD has increased by 31.7% from 2005 to 015 [8].

Chronic Kidney disease is also associated with huge economic burden. In high-income countries treatment of end-stage kidney disease (ESRD) shares more than 2–3% of their annual healthcare budget, while patients with ESRD represent only 0.03% of total population, and lower socioeconomic status is associated with greater risk of end-stage kidney disease [9]. In developing countries, the burden posed by CKD is much greater because of additional risks associated with poverty like: infections, hazardous work, poor education and poor maternal health combined with additional cost of screening and treatment where these costs have to be paid directly by patients [10].

Over half of all people requiring renal replacement therapy died due to a lack of access to dialysis or transplantation worldwide. Lack of access to renal replacement treatment in Africa, particularly middle and east Africa is the largest, where less than 3% of people requiring renal replacement therapy receive it [11]. As a result, people with end-stage kidney disease continue to die in the face of established treatment options and countries least equipped to provide dialysis or kidney transplantations are highly affected by the growing burden of CKD [12].

Early detection and treatment of chronic kidney disease can prevent or minimize complications associated with CKD [13]; however majority of CKD cases were not clinically recognized mainly because of the lack of patients' awareness about CKD and associated risk factors [14, 15]. It was conveyed that among 9772 adult patients admitted to a tertiary-care hospital, 40–70% of the patients were at risk for developing CKD [16]. Screening of patients at risk for CKD using objective measures found that 29% of those patients had CKD, though, only 7% of them were aware of having kidney disease [17].

Thus, assessment of knowledge, attitudes, and practices could be an early step forward to determine the extent to which an individual can follow healthy behaviors [18]. Screening Clinical indicators of renal dysfunction is fundamental for the early detection of patients at risk for CKD; moreover, it is also imperative to increase patients' awareness in order to modify their lifestyle towards preventing the occurrence of the disease. Early screening and population education regarding CKD positively affected patients' understanding of CKD and medical outcomes [19]. Therefore, the current study is aimed at assessing patient awareness, prevalence, and risk factors for CKD among diabetes mellitus and hypertensive patients.

## 2. Methods and Participants

### 2.1. Study Setting and Population

The study was conducted at Jimma university medical center, which is the only referral hospital for the south west region. Adult (age ≥ 18 years) diabetes mellitus and hypertensive patients on regular follow-up and those who signed informed written consent were recruited to the study. Confirmed chronic kidney disease, short follow-up period (< 3 months), incomplete patient chart, pregnant women, and critically ill patients were excluded from the study.

### 2.2. Study Design

Hospital based cross-section study design was used at JUMC to assess awareness, prevalence, and risk factors for chronic kidney disease among hypertensive and diabetic patients on regular follow-up. The sample size was calculated by using simple proportion formula with estimated prevalence of CKD among diabetes mellitus patients, p=18.1% [20], 95 confidence interval, and sample error of 5%, when adjusted for total number of source population, N =3,000, and final sample size, n≈ 208

### 2.3. Data Collection Procedures

Data collection tool was developed after reviewing different literatures; the tool has three parts: the first part is sociodemographic and disease condition, second part has 7 yes or no questions assessing patients knowledge, and third part has 6 questions assessing attitude; the tool was translated to Afan Oromo and Amharic languages. Using these tools patients were interviewed to obtain respective information when they come for monthly check-up and medication refill. Patient's chart was also reviewed for information like medications, blood pressure, and serum glucose level. The last three months' FBS (fasting blood sugar) and BP (blood pressure) average were used assess glycemic and blood pressure control, respectively.

Average knowledge is when at least 4 questions were answered correctly out of 7 knowledge questions, and positive attitude is when patients agreed with at least three of the attitude statements. Patient awareness was assessed using knowledge and attitude; it is considered as patients have awareness about CKD if they have both average knowledge and positive attitude.

Laboratory technologists drained 3cc of vein blood according to standard vein puncture, and the serum creatinine level and blood urea nitrogen were determined using ARCHITECT c8000 by kinetic alkaline picrate, and 10cc of middle stream urine was collected and urine dipstick was used to determine urine albumin level which was reported as negative, or +1, to +4). Glomerular filtration rate (GFR) was estimated using CKD –EPI question [21]; chronic kidney disease was defined using eGFR and presence of albuminuria and classified into five stages according to KDIGO classification system [2].

### 2.4. Ethics

The study protocol was approved by the institutional review board (IRB) of Jimma University, Institute of Health Sciences, and ethical clearance was obtained. Permission of data collection was granted with formal letter from chief executive director of JUMC. The purpose and protocol of this study was explained to participants and written informed consent was obtained from each patient. The privacy of personal information was protected and kept confidential. Codes were used for patient identification instead of names. Patients with abnormal findings were informed and referred to physicians for further investigation and appropriate management.

### 2.5. Data Analysis

Data was checked for completeness, grouped, then entered to EPI data, and exported to SPSS version 20 for analysis. Descriptive statistics like percentage and mean and standard deviation were used to present sociodemography and clinical characteristics of participants. Bivariate logistic regression was used to assess the crude association between independent variables and CKD and variables with* p-*value ≤ 0.25 were considered for multivariate logistic regression. Finally backward conditional logistic regression was used to identify independent predictors of CKD and* p*-value < 0.05 was considered statistically significant.

## 3. Result

A total of 208 hypertensive and diabetes mellitus patients were included in the study, of which 110 (52.9%) of the participants were male with mean (± SD) age of 54.81 ± 12.45 years. Majority of the study participants 145 (69.7%) were married, 142(68.7%) were rural dwellers, and 136 (65.4%) were educated at least up to elementary level (see Table 1).

The mean (±SD) systolic blood pressure and diastolic blood pressure of study participants were 136.6 ± 15.7 mmHg and 85 ± 8.9 mmHg, respectively. Mean ((±SD) fasting blood sugar was 145.6 ± 52 mg/dl and more than half 114 (54.8%) of the study participants had healthy body weight. Seventy-six (36.5%) of participants had average knowledge while 86 (41.3%) had positive attitude towards chronic kidney disease. Majority 143 (68.8%) of the study participants do not use social drugs like tobacco, alcohol, and Khat (see Table 2).

### 3.1. Patient Awareness of Chronic Kidney Disease

About 59 (28.4%) of the participants had awareness about CKD and 76 (36.5%) of the participants had average knowledge (see Table 2). Over half 109 (52.4%) of the study participants knew that CKD is reduced ability of the kidneys to avoid waste from the blood; only 80 (38.5%) and 92 (44.2%) of the respondents knew that hypertension and diabetes mellitus are risk factors for chronic kidney disease, respectively. Knowledge of the participants regarding progressive nature of CKD and kidney failure treatment cost was very low. Overall, 86 (41.3%) participants had positive attitude towards early detection and prevention of CKD; most 190 (72.1%) of the respondents prefer to go to health facility if they have sign of CKD and 150 (91.3) agreed that renal function test shall be done despite absence of symptoms; however majority 149 (71.6%) of the respondents believed renal function screening is costly (see Table 3).

### 3.2. Magnitude and Stages of CKD

The overall prevalence of chronic kidney disease was 26% with (95% CI, 20.3%-31.8%); of this, majority 41 (75.6) of the CKD were detected at early stage (normal or mildly decreased e- GFR with albuminuria), while 8 (14.8%) were with moderately increased risk for chronic kidney disease and 5 (9.3%) were high risk group (see Table 4).

### 3.3. Factors Associated with Chronic Kidney Disease

In crude analysis using bivariate logistic regression, age greater than 55 years, rural residence, ACEIs nonusers, poor CKD knowledge, negative attitude, long duration of hypertension, long duration of diabetes mellitus, social drug use, high level of fasting blood sugar, and uncontrolled blood pressure were associated significantly with chronic kidney disease.

Independent predictors for chronic kidney disease were identified using multivariate logistic regression; ACEIs nonusers, poor knowledge of CKD, long duration of hypertension, fasting blood sugar ≥ 150 mg/dl, uncontrolled blood pressure (>140/90mmHG) were found to be independent predictors of chronic kidney disease,

According to our finding, patients with uncontrolled blood pressure were 2.22 (AOR; 2.22 95% CI 1.01-4.76) times at risk for chronic kidney disease than those with controlled blood pressure. Patients who were not using angiotensin converting enzyme inhibitors (ACEIs) were 4.35 (AOR 4.35, 95% CI 1.96-10.0) times likely to develop chronic kidney disease when compared to users (see Table 5).

## 4. Discussion

In our study we assessed patient awareness (knowledge, attitude), prevalence of CKD, and its predictors among hypertensive and diabetes mellitus patients. Overall knowledge about CKD was low: only 36.5% of the participants have average knowledge which was similar with low knowledge level (mean=3.85) reported from community study in Tanzania [22], higher than what was reported from Nigeria where only 27% of study participants had good knowledge [23], but lower than that report from Jordan where 50% of the participants scored >80% correct scores [14]. This difference might be due to difference in health literacy of the study population or difference in number and type of questions used to assess patients' knowledge. Regarding knowledge of CKD risk factors only 38.5% and 44.2% of participants knew hypertension and diabetes mellitus can cause CKD, respectively; this was in line with finding in Nigeria [23], which was 38.3% and 43.6%, respectively, but higher than the report from Iran where only 12.7% of the respondents selected “unmanaged diabetes” and 14.4% selected “unmanaged hypertension” as “very likely to result in CKD” [24]. This disparity might be due to difference in study setting community versus facility based and the tools used were different. We used dichotomous question while it was Likert scale in the study from Iran.

Overall attitude favoring early detection and prevention of CKD was low; most 149 (71.6%) of the participants believed kidney function screening is costly, though majority 190 (90.3%) of the respondents agreed to seek modern medical care; this was in agreement with report from Tanzania. All (100%) were willing to seek healthcare from a biomedical clinic and many participants with CKD were concerned about the health and economic impact of a diagnosis of kidney disease [22].

The prevalence of chronic kidney disease was found to be 26% (95% CI:20.07%- 31.93%) which was corresponding to the report from UK which was 27.5% [25] higher than previous reports from similar setting, 18.1% from southern part of Ethiopia [21] and 20.8% from Gondar [26], but lower than the prevalence reported from Spain where the prevalence of CKD among both diabetic and hypertensive patients was 31.22% [27] and prevalence of CKD among type-2 DM in USA [28]. These discrepancies might be due to difference in the study population; in the first two studies the study population was diabetic patients, while it might be due to studying setting difference and large sample size used in Spain and USA studies.

In our study, fasting blood sugar>150mg/dl was independently associated with chronic kidney disease (AOR; 3.70, 95% CI; 1.75-7.69); this was similar with report from USA were high level of Hemoglobin A1c was associated with CKD [28], and in southern Ethiopia uncontrolled diabetes mellitus was independently associated with CKD. [20], while it was only associated with bivariate logistic regression according the study from Gondar [26].

This study also revealed that long duration of hypertension and uncontrolled blood pressure (BP> 140/90 mmHg) were independent predictors of CKD; this supports previous reports, where uncontrolled blood pressure was associated with high risk of CKD [20, 28] and among the participants at risk for CKD, 113 (83.0%) had poorly controlled hypertension [22]. It was also in line with earlier findings, where long-term, uncontrolled, high blood pressure was found to be initiating factor for high intraglomerular pressure, leading to impairing glomerular filtration, microalbuminuria, or proteinuria [29, 30].

Use of ACEIs in the management of hypertension was found to be protective in the current study (AOR; 4.35, 95% CI; 1.96-10) which supported previous studies [31] even though the study populations are not the same. Patient knowledge was independently associated (AOR; 3.69, 95% CI 1.48-9.20) with chronic kidney disease. This was similar with report from Palestine which showed higher total knowledge score was associated with CKD prevention (p < 0.001) [32].

Age was significantly associated with bivariate logistic regression however not significantly associated with the final analysis which was different from most findings [20, 24, 26]. This difference might be due to small sample size of the current study. Other factors like duration of diabetes mellitus and place of residence were significant in the crude analysis only, while sex, family history of kidney disease, body mass index were not significantly associated.

Our study used cross-sectional study design; temporal relationship between risk factors and the outcome cannot be determined. Single measurement of serum creatinine and urine albumin was used to define CKD; semiquantitative urine albumin was used and so could not grade level of albuminuria, and the assessment of knowledge and attitude was based on self-report with possible risk of bias; we used “yes” or” no” and “agree and disagree” type of questions which limit possible answer of the respondents.

Despite these limitations, the finding of this study can be used as input for researchers and healthcare providers because as to our knowledge there was no prior study assessing patient awareness of CKD and risk factors among patients with both hypertension and DM; it will greatly contribute to increasing awareness of chronic kidney disease and its risk factors.

## 5. Conclusion

Our study found out low level of patient awareness of chronic kidney disease and its risk factors among patients with both hypertension and diabetes mellitus. The prevalence of chronic kidney disease was high and uncontrolled blood pressure, high fasting blood sugar, long duration of hypertension, ACEIs nonusers, and poor knowledge about CKD were found to be independent predictors for chronic kidney disease. Working toward increasing patient awareness and screening for CKD at regular intervals among high risk groups is paramount to recognize chronic kidney disease at early stage before it progresses to kidney failure.

## Figures and Tables

**Table 1:** Sociodemographic characteristics of diabetes mellitus and hypertensive patients at JUMC.

Characteristics	Number	Percent
Age (year)		
<55	101	48.6
≥55	107	51.4

Sex		
Male	110	52.9
Female	98	47.1

Marital status		
Married	145	69.7
Single	63	30.3

Educational level		
No education	72	34.6
Elementary School	96	46.2
Secondary school	28	13.5
College and above	12	5.8

Average monthly income (ETB)		
<1000	117	56.2
1001-3000	37	17.8
3001-5000	34	16.3
Above 5000	20	9.6

Source of health information		
Health care providers	151	72.6
Television and Radio	49	23.6
Others	8	3.8

Residence		
Urban	142	68.3
Rural	66	31.7

ETB: Ethiopian Birr.

**Table 2:** Knowledge, attitude, and clinical characteristics of diabetes mellitus and hypertensive patients at JUMC.

Characteristics	Number (Mean	Percent (standard deviation)
Systolic pressure	136.6	±15.7
Diastolic pressure	85.6	±8.9
Fasting blood sugar	145.6	±51.2
Blood urea nitrogen	26.6	±13.2

Urine albumin		
Positive	51	24.5
Negative	157	74.5

BMI category (Kg/m^2^)		
Underweight (<18.5)	14	6.7
Healthy weight 18.5-24.9)	114	54.8
Overweight (25-29.9)	63	30.3
Obese (>30)	17	8.2

Social drug use		
Users	65	31.2
Non-Users	143	68.8

Knowledge about CKD		
Average knowledge	76	36.5
Poor knowledge	132	63.5

Attitude toward CKD		
Positive attitude	86	41.3
Negative attitude	122	58.7

Duration of hypertension (year)		
0-5	92	44.2
6-10	74	35.6
Above 10	42	20.2

Duration of DM		
0-5	90	43.3
6-10	80	38.5
Above 10	38	18.3

Family Hx of Kidney disease		
Positive	42	20.2
Negative	166	79.8

CKD: chronic kidney disease, BMI: body mass index, DM: Diabetes mellitus, Family Hx: family history.

**Table 3:** Knowledge and attitude of DM and hypertensive patients about CKD at JUMC.

Knowledge Questions	Response, Number (%)	
	Yes	No

CKD is reduced ability of the kidneys to avoid waste from the blood	109(52.4)	99(47.6)
Chronic kidney disease may not have any symptom until advanced	62(29.8)	146(70.2)
High blood pressure can cause chronic kidney disease	80(38.5)	128(61.5)
Diabetes mellitus can cause Chronic kidney disease	92(44.2)	116(55.8)
Chronic kidney disease progresses to kidney failure	40(19.2)	168(80.8)
Kidney failure is fatal if not treated by dialysis or kidney transplant	52(25)	156(75)
Kidney failure treatment costs more than kidney function screening	20(9.6)	188(90.4)

Attitude questions	Agree	Disagree

Kidney function test is necessary though there is no sign of CKD	150(72.1)	58(27.9)
It is not too expensive to have a kidney screening test.	59(28.4)	149(71.6)
Chronic kidney disease carries high risk of death	98(47.1)	110(52.9)
I will go to a health facility if I have signs of kidney disease	190(91.3)	18(8.7)
It is possible to prevent chronic kidney disease	120(57.7)	488(2.3)
Early detection of CKD is important to slow its progress	162(77.9)	46(22.1)

**Table 4:** Prevalence and stage of chronic kidney disease of diabetes mellitus and hypertensive patients at JUMC.

Stage of CKD	Description	eGFR (ml/min/1.73m2)	Number (%)
G1	Normal or high with albuminuria	≥90	13(6.25)
G2	Mildly decreased with albuminuria	60-89	28(13.5)
G3a	Mildly to moderately decreased	45-59	8(3.8)
G3b	Moderately to severely decreased	30-44	5(2.4)
G4	Severely decreased	15-29	0
G5	Kidney failure	<15	0
Total			54(26)

eGFR: estimated glomerular filtration rate using CKD-EPI equation.

**Table 5:** Independent variables associated with CKD among diabetes mellitus and hypertensive patients at JUMC.

Variable	CKD		Bivariate analysis		Multivariate analysis	
	Yes, N (%)	NO N (%)	COR (95% CI)	P-value	AOR (95%CI)	P-value
Sex:						
Female	24(11.5)	74(35.6)	1			
Male	30(14.4)	80(38.5)	1.12(0.62-2.12)	0.65		

Age(years)						
<55	19(9.1)	82(39.4)	1			
>/=55	35(16.8)	72(34.6)	2.08 (1.10-4.00)	0.02		

Marital status						
Married	37(17.8)	108(5.2)	0.93 (0.47-1.81)			
Single	17(8.2)	46(22.1)	1	0.83		

Family Hx of kidney disease						
Absent	32(15.4)	122(58.7)	1			
Present	10(4.8)	44(21.2)	0.87(0.39-1.91	0.72		

Residence:						
Urban	31(14.9)	111(54.1)	1			
Rural	23(11.6)	4320.7)	1.92(1.01-3.70)	0.048		

ACEIs/ARBS						
Users	29(13.9)	128(61.5)	1	<0.001	1	
Non users	25(12.0)	26(12.5)	4.17(2.13-8.33)		4.35(1.960-10.0)	<0.001

CKD knowledge						
Average	8(3.8)	69(33.2)	1		1	
Poor	46(22.1)	85(40.9)	4.67 (2.07-10.55)	<0.001	3.69(1.48-9.20)	0.005

Attitude toward CKD						
Positive	12(5.8)	74(35.6)	1			
Negative	42(20.2)	80(38.5)	3.24(1.58-6.62)			

Social drug use						
Non users	30(14.6)	113(54.3)	1			
Users	24(11.7)	41(19.7)	2.22(1.16-4.17)	0.016		

Duration of hypertension						
≤ 5 years	18(8.8)	74(36.1)	1	0.007	1	0.007
6-10 years	23(11.2)	57(27.4)	1.23(0.58-2.59)	0.59	3.03(1.18-7.69)	0.02
>10 years	13(6.3)	23(11.1)	3.39(1.53-7.53	0.003	4.55(1.72-11.11)	0.002

Duration of DM						
≤5 years	18(8.8)	72(34.6)	0.48(0.21-1.12	0.19		
6-10 years	17(8.2)	57(27.4)	0.78(0.34-1.77)	0.09		
>10 years	19(9.1)	25(12.2)	1	0.55		

Fasting blood sugar						
≤ 150 mg/dl	24(11.5)	110(52.9)	1	<0.001	1	
>150mg/dl	30(14.4)	44(21.2)	3.13(1.64-5.88)		3.70(1.75-7.69)	0.001

Blood pressure control						
Controlled	31(14.9)	119(58.0)	1		1	
Uncontrolled	22(10.6)	35(16.8)	2.44(1.23-4.23)	0.009	2.22(1.01-4.76)	0.046

ACEIs/ARBs: Angiotensin converting enzyme inhibitors/angiotensin receptor blockers, family Hx: family history.

## Data Availability

The data used to support the findings of this study are available from the corresponding author upon request.
